# Advances in enzyme-mediated proximity labeling and its potential for plant research

**DOI:** 10.1093/plphys/kiab479

**Published:** 2021-10-18

**Authors:** Andrea Mair, Dominique C Bergmann

**Affiliations:** Howard Hughes Medical Institute and Department of Biology, Stanford University, Stanford, California 94305, USA

## Abstract

Cellular processes rely on the intimate interplay of different molecules, including DNA, RNA, proteins, and metabolites. Obtaining and integrating data on their abundance and dynamics at high temporal and spatial resolution are essential for our understanding of plant growth and development. In the past decade, enzymatic proximity labeling (PL) has emerged as a powerful tool to study local protein and nucleotide ensembles, discover protein–protein and protein–nucleotide interactions, and resolve questions about protein localization and membrane topology. An ever-growing number and continuous improvement of enzymes and methods keep broadening the spectrum of possible applications for PL and make it more accessible to different organisms, including plants. While initial PL experiments in plants required high expression levels and long labeling times, recently developed faster enzymes now enable PL of proteins on a cell type-specific level, even with low-abundant baits, and in different plant species. Moreover, expanding the use of PL for additional purposes, such as identification of locus-specific gene regulators or high-resolution electron microscopy may now be in reach. In this review, we give an overview of currently available PL enzymes and their applications in mammalian cell culture and plants. We discuss the challenges and limitations of PL methods and highlight open questions and possible future directions for PL in plants.

## Teaser

Innovation of new enzymes and methods makes proximity labeling an attractive tool for discovery of protein interactions and subcellular proteomes and broadens the spectrum of applications in plants.

## Introduction

Continuous development of technologies to query cellular contents advances our understanding of plant growth and development. In the face of changing and increasingly challenging global plant growth conditions, it is more important than ever to capture and integrate multi-level omics and interaction data. The ambitious “plant cell atlas” project is a community effort to build a resource for integrated tissue- to single-cell- and molecule-level nucleotide, protein, and metabolite data (Rhee et al., 2019). While nucleotide-centric methods like single-cell RNA-sequencing (scRNA-seq) and single nucleus ATAC-seq are gaining traction in plants (Farmer et al., 2021; Rich-Griffin et al., 2020), single-cell proteome or protein interactome mapping is still more challenging and standard methods for exploring protein-centric data have clear limitations. For example, identification of subcellular proteomes by fractionation is limited to compartments that can be purified and often suffers from contamination with other organelles. Protein–protein interactions (PPIs) are traditionally explored by methods such as yeast two-hybrid, peptide, or protein arrays or affinity purification coupled to mass spectrometry (AP-MS). These techniques are prone to produce false positives from artificial co-expression, co-localization after cell lysis, or from crosslinking and false negatives due to missing scaffolds or co-factors. Furthermore, AP-MS often fails to identify transient or weak interactions and interactions with membrane-localized or unstable proteins.

**Figure 1:**
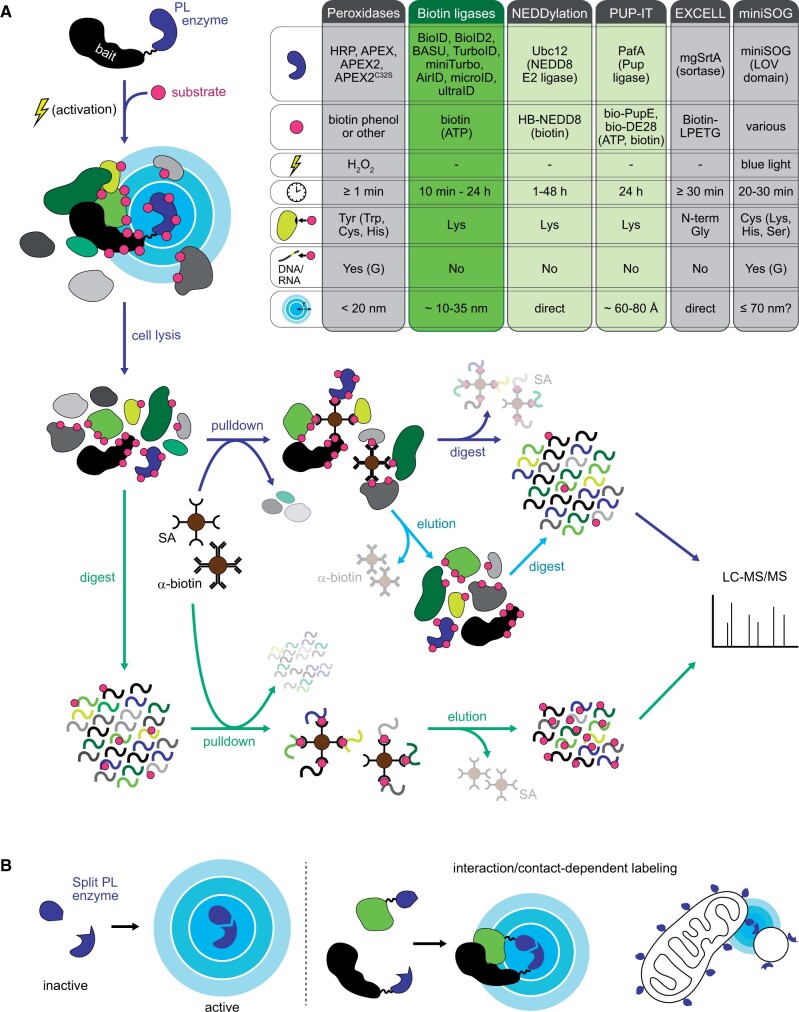
Overview of enzymatic PL and characteristics of different enzymes. A, General principle of PL: the scheme on the left shows typical steps of a PL experiment for protein discovery by MS: the PL enzyme is fused to a bait protein or subcellular targeting sequence. Upon addition of a biotin substrate and activation of the enzyme (if required), nearby proteins are labeled. Cells are lysed and proteins can be isolated using streptavidin beads, followed by on-bead digest and MS analysis (dark blue arrows). Alternatively, biotinylated proteins can be purified with biotin-specific antibodies, eluted and digested for MS (light blue arrows), or proteins can be digested before peptide-level pulldowns with streptavidin beads for enrichment of biotinylated peptides (green arrows). The table on the right summarizes available PL enzymes as well as experimental requirements and characteristics of each enzyme class (top to bottom: enzyme variants, substrates, activation requirements, labeling time, target amino acids on proteins, and bases on DNA/RNA, and estimated labeling radius). Box colors indicate suitability for use in living plants (dark green = used, light green = might be adapted, gray = currently not suitable). For more details, see Supplemental Table S1. B, Principle (left) and examples for application (right) of split-PL enzymes. The PL enzyme is split into two inactive halves that regain activity when brought into close proximity, for example, in the context of PPI or at organelle and/or membrane contact sites.

In the past decade, enzyme-catalyzed proximity labeling (PL) has emerged as a useful alternative tool for mapping of PPIs and cellular proteomes and transcriptomes. PL generally involves transformation of an inert substrate into a short-lived reactive species that covalently attaches to nearby proteins or nucleotides for easy visualization and stringent purification without the need for cell fractionation or crosslinking (Figure  1). PL, therefore, avoids many of the challenges of traditional methods.

Here, we give an overview over currently available PL enzymes and their applications. We highlight molecules and methodologies currently working in or likely adaptable for plants, summarize past plant PL studies, discuss general and specific challenges, and list useful resources for conducting successful PL experiments.

## Enzyme-based PL methods

Enzymes adapted for use in PL can roughly be classified into three groups (peroxidases, biotin ligases, and other enzymes). They differ in their substrates, kinetics, labeling radius, ideal labeling conditions, and applicability in different systems and organisms (Figure  1;Supplemental Table S1).

### Peroxidases

The first group of enzymes is plant heme peroxidases that have been adapted for PL and protein localization through electron microscopy (EM). Peroxidases can be used with a wide range of substrates, including biotin-phenol and other phenol derivatives. Upon activation by hydrogen peroxide (H_2_O_2_), the phenol substrate is turned into an extremely short-lived (<1 ms) phenoxyl radical, which covalently attaches to electron-rich amino acids (Tyr and to a lesser extent Trp, His, and Cys) on proteins (Rhee et al., 2013) and guanosine in RNA and DNA molecules (Fazal et al., 2019; Padron et al., 2019; Zhou et al., 2019) in a <20 nm radius.

The first of these, horseradish peroxidase (**HRP**), can be fused to a protein of interest (POI) or targeted with commercial HRP-conjugated antibodies or ligands (Kotani et al., 2008; Rees et al., 2015). HRP use is limited to cell surfaces and compartments with oxidizing conditions like the secretory pathway or the ER, since its structural integrity depends on disulfide bonds and calcium (Ca^2+^)-binding (Go and Jones, 2008; Martell et al., 2012). **APEX**, an activity-enhanced version of pea (*Pisum sativum*) or soybean (*Glycine max*) L-ascorbate peroxidase (Martell et al., 2012; Rhee et al., 2013), and its derivatives do not suffer from this limitation and function in many cellular compartments (Fazal et al., 2019). **APEX2**, which was derived from (soybean) APEX by yeast display-based evolution, has improved activity and sensitivity, allowing use with low expressing bait proteins (Lam et al., 2015) and **APEX2^C32S^**, which lacks a conserved Cys residue, promises improved stability of APEX2 fusion proteins (Huang et al., 2019).

Major advantages of peroxidase-based labeling are an extremely high labeling speed (1 min with APEX/APEX2) and the ability to timely control the labeling process through H_2_O_2_ availability. Cellular toxicity of H_2_O_2_ and poor membrane permeability of biotin-phenol, however, have limited the use of APEX methods primarily to cultured human cells.

### Biotin ligases

A second group of PL enzymes is derived from the bacterial biotin ligase biotin retention A (BirA), which uses ATP to activate and covalently attach biotin to a Lys residue on acetyl-CoA carboxylase (Barker and Campbell, 1981; Rodionov et al., 2002). The extraordinary strength of the biotin–avidin interaction allows for stringent purification of biotinylated macromolecules with streptavidin and is a strong appeal of PL.

**BirA** was first used for protein purification, chromatin immunoprecipitation (ChIP), and cell surface labeling by engineering a biotin acceptor domain onto the target and co-expressing the ligase (Duffy et al., 1998; de Boer et al., 2003; Viens et al., 2004; Howarth et al., 2005). While it is still used for some methods (Deal and Henikoff, 2011; Jan et al., 2014; Williams et al., 2014), its potential for explorative purposes is quite limited.

To make the protein more versatile for PL, promiscuous versions of BirA (BirA*) were engineered, which release activated biotin to diffuse away and covalently attach to primary amines of proximal proteins (Lys and protein N-termini). The labeling radius was estimated to be about 10 nm using the nuclear pore complex as a molecular ruler (Kim et al., 2014), but may vary depending on the subcellular compartment (Choi-Rhee et al., 2004), biotin ligase version (Branon et al., 2018; May et al., 2020), and length of the flexible linker between bait and ligase (Kim et al., 2016).

There is a continuous effort to find new and improved versions of BirA*. The original proximity-dependent biotin identification (**BioID**) using *Escherichia* *coli* BirA* (R118G; Choi-Rhee et al., 2004; Cronan, 2005; Roux et al., 2012) is relatively slow and requires labeling times of ∼24 h. **BioID2**, generated from *Aquifex aeolicus* BirA (Kim et al., 2016), is smaller and requires less biotin, but has an increased temperature optimum (50°C). Modified *Bacillus subtilis* BirA (**BASU**) was reported to have improved labeling speed in one study (Ramanathan et al., 2018), but not in others (Branon et al., 2018). Using yeast display-based directed evolution, Branon et al. (2018) engineered two highly active versions of *E. coli* BirA (**TurboID** and **miniTurbo**), reducing labeling times to as little as 10 min and broadening the optimal temperature range and accessibility to different model organisms. High activity of TurboID, however, comes at the cost of a slightly bigger labeling radius (≥35 nm, increasing with time; May et al., 2020), low-level activity with endogenous biotin and cellular toxicity (Branon et al., 2018). Compared to TurboID, miniTurbo is half as active, but has lower background in the absence of exogenous biotin (Branon et al., 2018; Mair et al., 2019; Zhang et al., 2019). **AirID**, a synthetic BirA* based on ancestral enzyme reconstruction from metagenome data, also shows greatly enhanced activity (1–6 h labeling time) and a broader temperature range (Kido et al., 2020). The most recent and smallest BirA* variants are **microID** and **ultraID** (Zhao et al., 2021), which were engineered from BioID2 and have kinetics similar to TurboID in human cells with less background labeling from endogenous biotin.

A major advantage of biotin ligase-based methods is that labeling only requires the addition of biotin, which is membrane-permeable and nontoxic. As a result, biotin ligases have been used in many different systems, including live animals and plants. The long labeling times, baseline activity in the presence of endogenous biotin, and inability to tightly control enzyme activity by activation/inactivation, however, result in poor control over labeling timing. This makes biotin ligases less suitable for experimental questions requiring short and precisely timed labeling and increases stochastic (nonspecific) “background” labeling. Approaches to circumvent issues caused by constant activity of BirA* include the use of Split-BioID variants (see below) and a two component BioID (2C-BioID; Chojnowski et al., 2018). BioID is also less versatile than APEX, with labeling being limited to proteins and overall fewer methods being currently available.

### Other PL enzymes

Several other enzymes and processes are being modified for PL methods. These include **NEDDylation**, where fusion of the human NEDD8 (developmentally down-regulated protein 8) E2 ligase Ubiquitin (Ubq)-conjugating 12 (Ubc12) to a POI or small molecule directs addition of Ubq-like NEDD8 to Lys residues of interacting proteins (Zhuang et al., 2013; Hill et al., 2016; Chu et al., 2020). By adding a His_6_-biotin tag to NEDD8 (HB-NEDD8), which can be biotinylated by BirA (Zhuang et al., 2013) or endogenous biotin ligases (Chu et al., 2020), the NEDD8-tag enables dual affinity purification for increased specificity. Although fast in cell lysates, in vivo NEDDylation may require long labeling times (Chu et al., 2020). Another method, called pupylation-based interaction tagging (**PUP-IT**; Liu et al., 2014), employs the promiscuous bacterial prokaryotic Ubq-like protein (Pup) ligase proteasome accessory factor A (PafA) to conjugate the small protein Pup to Lys residues on nearby proteins in the presence of ATP. Adding a carboxylase domain, which is biotinylated by endogenous biotin ligases, to Pup, allows for efficient purification with streptavidin beads. Since Pup does not diffuse across membranes, this method is likely not suitable for labeling PPIs in all organelles. Two additional methods/enzymes, enzyme-mediated proximity cell labeling (**EXCELL**; Ge et al., 2019) and mini Singlet Oxygen Generator (**miniSOG**), are less useful for plant applications. EXCELL uses the enzyme monoglycine *Staphylococcus aureus* transpeptidase sortase A to covalently link a small peptide (Biotin-LPXTG) to proteins, but the requirement of an N-terminal Gly on prey proteins makes EXCELL unsuitable for explorative PPI studies. MiniSOG generates singlet oxygen, which activates labeling probes that covalently bind proteins (To et al., 2016), RNA (Wang et al., 2019b), or DNA (Ding et al., 2020) and can also be used for microscopy (Shu et al., 2011; Ng et al., 2016; Boassa et al., 2019), but is light-activated.

### Split enzymes for PL

Split-PL enzymes can be used for identification of context-specific local proteomes and protein complexes and allow for tighter labeling control (Choi-Rhee et al., 2004; Kwak et al., 2020). Similar to bimolecular fluorescence complementation(BiFC), the PL enzyme is split into two catalytically inactive parts, which can be fused to two different POIs or targeting sequences and reconstitute into an active enzyme when brought close together (Figure  1). Especially for BioID methods, the use of split enzymes can substantially reduce background labeling and add temporal control. By fusing the PL enzyme halves to FK506-binding protein (FKBP) and FKBP-rapamycin-binding protein (FRB), which interact in the presence of rapamycin, labeling can further be made inducible (Schopp et al., 2017; Xue et al., 2017; Han et al., 2019; Cho et al., 2020a). Using appropriate receivers, other triggers such as developmental cues or cellular signals such as Ca^2+^ could also be used.

Split versions are available for many PL enzymes: HRP (Martell et al., 2016), APEX2 (Xue et al., 2017; Han et al., 2019), BioD (De Munter et al., 2017; Schopp et al., 2017; Kwak et al., 2020), TurboID (Cho et al., 2020a, 2020b), and miniSOG (Boassa et al., 2019). Their activity, however, is generally much lower than that of their whole counterparts, which increases labeling time. For example, split-TurboID requires 4 h to reach labeling levels comparable to 1 min with full-length TurboID.

Another caveat of split PL systems is that the protein halves need to be able to get close together. Since protein conformation and linker length in the fusion proteins can influence the ability of the enzyme to reconstitute, constructs should be tested prior to use.

## A brief history of using PL techniques in plants

So far, all PL techniques successfully applied in plants are biotin ligase-based methods (see Supplemental Table S2). Pioneering early work (Lin et al., 2017), used the maize (*Zea mays*) UBQ promoter to express rice (*Oryza sativa*) FD transcription factors 1 and 2 (OsFD1 and OsFD2), fused to BirA* with a cryptic splice site removed (BirAG), in rice protoplasts and identified several putative interactors. Later studies used BioID to gain insight into plant–pathogen interactions using transient expression with the 35S promoter in *Nicotiana benthamiana* (Conlan et al., 2018; Das et al., 2019; Macharia et al., 2019) or dexamethasone-inducible overexpression in stable Arabidopsis lines (Khan et al., 2018). Recently, Tang et al. (2020) and Huang et al. (2020) tagged multiple proteins of the nuclear membrane and nuclear pore complex with BioID2, under the 35S promoter in stable Arabidopsis lines and identified (trans)membrane proteins specific for the outer and inner nuclear membrane, as well as components of the inner nuclear membrane protein degradation machinery.

Introduction of the faster variants TurboID and miniTurbo broadened the spectrum of addressable biological questions substantially. In 2019 and 2020, several groups showed that TurboID and miniTurbo work in different plant systems and enable efficient labeling in a much shorter time frame, even at low (cell type-specific) expression levels (Mair et al., 2019; Zhang et al., 2019, 2020a; Arora et al., 2020). Zhang et al. (2019) compared activities of BioID, BioID2, and TurboID in *N. benthamiana* and used the Toll-interleukin-1-receptor-nucleotide-binding leucine-rich repeat immune receptor N, expressed under the 35S promoter, to identify mediators of effector-triggered immunity. Mair et al. (2019) showed high activity of both TurboID and miniTurbo compared to BioID in *N. benthamiana* and in numerous different Arabidopsis tissues when expressed with the Arabidopsis UBQ10 promoter. Mair et al. (2019) also used the stomatal lineage-specific transcription factor FAMA to demonstrate the applicability of these PL enzymes for identifying protein complexes of low abundant or rare proteins and for probing cell-type-specific organellar proteomes. Finally, Arora et al. (2020) conducted a systematic comparison of the activity of BioID, BioID2, TurboID, and miniTurbo expressed under the 35S promoter in tomato (*Solanum lycopersicum*) hairy root culture, *N. benthamiana* leaves, and Arabidopsis cell culture. Arora et al. (2020) further used the Arabidopsis TPLATE complex to test the effect of different linker lengths on labeling of the complex components and to establish a stringent and combined protocol for recovery of nonbiotinylated and biotinylated peptides. In all three studies, TurboID (and miniTurbo) were found to have high activity at plant growth temperatures (22–28°C). Labeling times of 5–15 min were sufficient to detect some labeling, but longer labeling of 1–12 h was found beneficial for identification of labeled proteins by MS and to allow for plant response to pathogen treatment. In addition to these studies, Kim et al. (2019) identified interactors of BRASSINOSTEROID-INSENSITIVE 2 (BIN2), a negative regulator of brassinosteroid signaling, and provided an alternative protocol for purification of biotinylated peptides. The experimental conditions and protocols established by these first TurboID studies provide helpful guidelines and tools for future PL studies. Most recently, Xu et al. (2021) used TurboID to identify TPR (TOPLESS) and TPR-related proteins as specific cargo of the nuclear exportin EXPORTIN-4, which plays a role in effector-triggered immunity.

## Oh, the possibilities!—applications for enzymatic PL

### PPIs and protein networks

**Figure 2:**
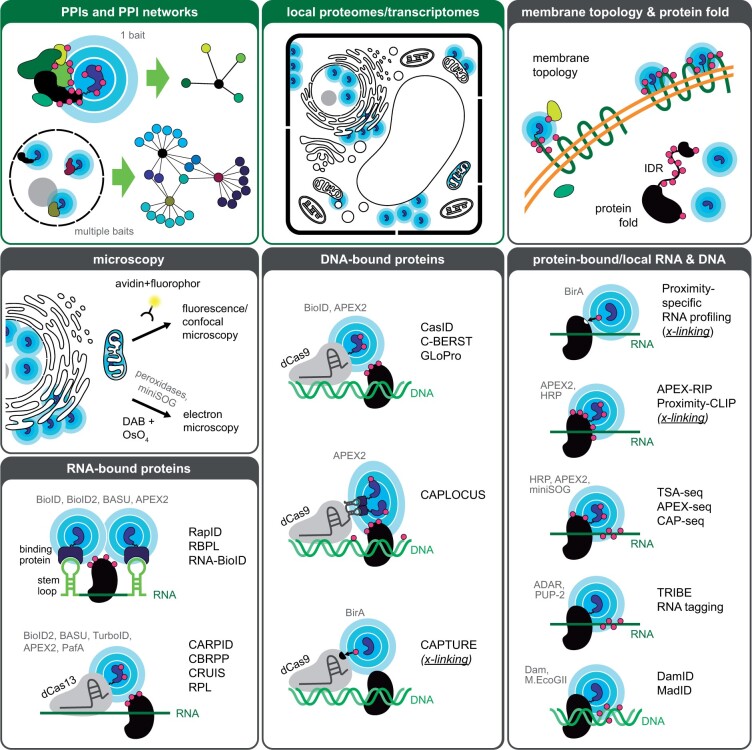
Applications for enzymatic PL. PL methods have been developed for a multitude of applications, including discovery of PPIs, local proteomes and transcriptomes, membrane topology and fold of proteins, microscopy, identification of RNA/DNA-bound proteins and of local (e.g. in organelles) and protein-bound RNA and DNA sequences. So far, only the first two have been used in plants (green boxes), but others may be adaptable with some modifications (see main text). For more details on DNA/RNA methods and plant experiments, see Supplemental Tables S2 and S3.

One major application for PL is to identify putative PPIs and build protein interaction networks (Figure  2). Since PL can be done in planta under close to natural conditions, some false positives (e.g. from artificial co-expression) and negatives (e.g. from missing co-factors or scaffolds) associated with traditional methods can be avoided. Moreover, weak and transient interactions, as well as proteins that need harsh/denaturing conditions for purification, such as membrane proteins, can be captured. PL is also superior for identification of putative interactors of proteins that are low abundant and expressed in a timely and/or spatially restricted manner (Mair et al., 2019). By increasing the linker length (Kim et al., 2016; Arora et al., 2020), using multiple baits or by implementing an iterative PL strategy, larger protein complexes and protein interaction networks can be studied. Split-PL approaches further enable protein complexes to be captured in a spatially and/or temporally controlled manner and dissection of distinct complexes a single protein may engage. One possibility that needs to be explored further is whether looking at the distribution of biotinylated peptides along prey proteins can reveal protein complex conformation, as was suggested by Arora et al. (2020).

### Local proteomes

Another popular application for PL is the identification of local proteomes, including whole organelles/cellular compartments, cellular domains, and microdomains (Figure  2). PL does not require these compartments to be purified, which broadens the spectrum of possible targets and can reduce contamination (e.g. from chloroplasts) substantially. This approach has been used to characterize the proteomes of individual organelles, membrane domains, and lipid droplets among others (for examples see: Mick et al., 2015; Branon et al., 2018; Cijsouw et al., 2018; Bersuker and Olzmann, 2019; Mair et al., 2019; Tang et al., 2020). Most recently, Go et al. (2021) built a BioID-based proteome atlas of human cells using 234 baits in 32 cellular compartments. Beyond catching a steady state glimpse of bulk organelles, PL could also be used to study local proteomes in a cell type-specific or treatment-dependent way to identify unique protein compositions and gain insight into protein dynamics. The use of split-PL approaches again enables further spatial or temporal specification and can be used, for example, to study membrane contact sites (Kwak et al., 2020; Cho et al., 2020a).

### Protein topology and fold

For integral membrane proteins, it is often unclear how they are inserted into the membrane. PL, in combination with microscopy or MS, can be used to address this question. For example, when targeting the PL enzyme to one end of the POI, protein topology can be inferred from the subcellular distribution of the biotinylation signal or from labeled proteins with known subcellular localization. Alternatively, when the PL enzyme is targeted to one face of the membrane, biotinylated peptides of the POI itself can provide information on its orientation and compartment-exposed areas (Figure  2) (Martell et al., 2012; Kwak et al., 2020). Similarly, PL and identification of biotinylated peptides can be used to get an idea of the protein fold (surface exposed versus buried amino acids) and the order/disorder state of a protein (Figure  2). A study by Minde et al. (2020) found a clear bias toward labeling of intrinsically disordered regions (IDRs) in proteins, which are generally more accessible.

### Microscopy

PL enzymes are also regularly used in combination with microscopy techniques, including fluorescence microscopy (for confirming localization and labeling efficiency) and EM (for high-resolution information on protein/PPI localization and topology) (Figure  2). In addition to antibody/immune-gold labeling of biotinylation, which can be applied to both peroxidase and biotin ligase methods, HRP/APEX/APEX2 and miniSOG can be used in combination with DAB (3,3′-diaminobenzidine) to generate high contrast labeling for EM. When activated, DAB polymerizes into local (≤20 nm) precipitates that can be visualized by light microscopy (Xue et al., 2017) and EM after treatment with OsO_4_ (Shu et al., 2011; Martell et al., 2012).

Many sample preparation methods have been developed for PL-assisted light and electron microscopy (Martell et al., 2017; Tsang et al., 2018; Sengupta et al., 2019; Rae et al., 2020). Notably, since labeling happens in fixed tissue, plants are amenable to DAB staining (Graham and Karnovsky, 1966; Ekes, 1971) and methods for (cryo)EM, cryo-electron tomography, and correlated light and electron microscopy (CLEM) are generally accessible to plant tissues (Otegui and Pennington, 2019; Wang et al., 2019a), some APEX-cryoEM methods (Tsang et al., 2018; Sengupta et al., 2019) hold promise for plant use.

### Identification of proteins binding specific DNA/RNA sequences

A multitude of protocols have been developed to target PL enzymes to specific DNA or RNA sequences for labeling of proximal proteins (see Figure  2;Supplemental Table S3 for details on used PL enzymes and targeting strategies).

DNA-centric PL methods use guide RNAs to target dCas9-fused PL enzymes to specific sequences and can be used to study local chromatin compositions and architecture and to identify transcriptional and epigenetic regulators of specific genes. These methods include CasID (Schmidtmann et al., 2016), CAPTURE (Liu et al., 2017), C-BERST (Gao et al., 2018), GLoPro (Myers et al., 2018), and CAPLOCUS (Qiu et al., 2019). Sufficient enzyme density at the locus of interest can be achieved through tiling guide RNAs (Myers et al., 2018), which also reduces noise from off-target binding, or by adding multiple MS2 stem loops to the guide for signal amplification, which are bound by PL enzyme-fused MS2 coat proteins (Qiu et al., 2019). By including a crosslinking and sonication step, noncoding RNAs and long-range DNA interactions can also be captured (Liu et al., 2017; Qiu et al., 2019).

RNA-centric PL methods (RapID; Ramanathan et al., 2018), RBPL (Lu and Wei, 2019), RNA-BioID (Mukherjee et al., 2019; Han et al., 2020), CARPID (Yi et al., 2020), CBRPP (Li et al., 2021b), CRUIS (Zhang et al., 2020b), and RPL (Lin et al., 2020) can be used to study dynamic interactomes of different RNA species and to identify regulators of RNA activity, localization, and stability. For targeting, they employ either stem loops, which are added to the RNA of interest and bound by the corresponding viral coat proteins or dCas13 and sequence-specific guide RNAs (Figure  2). The latter allows targeting of endogenous RNAs, but can suffer from the size (∼120 kDa) and lower sensitivity of dCas13 (Han et al., 2020).

While the speed of APEX2-based methods allows for improved analysis of dynamics in chromatin states and RNA interactomes, BirA-based methods (CasID, CAPTURE, RapID, RBPL, RNA-BioID, CARPID, and CBRPP) will be accessible to a broader range of model organisms. At least some of these should be adaptable for use in plants.

### Identification of protein-bound DNA/RNA

Commonly used techniques for identification of protein-bound or local DNA and RNA sequences include ChIP-seq, RIP-seq, cross-linking immunoprecipitation (CLIP)-seq, fluorescence in situ hybridization microscopy, and Fractionation-seq. To circumvent some of the limitations of these methods (e.g. requirement for crosslinking and/or specific antibodies and ability to purify the organelle of interest), diverse PL methods for DNA and RNA discovery have been developed (see Figure  2;Supplemental Table S3 for details on used PL enzymes and targeting strategies).

Crosslinking methods for RNA discovery generally rely on biotin labeling of proteins for purification. RNAs that are bound or proximal to the POI are identified by crosslinking to and co-affinity purification with the POI, followed by decrosslinking and sequencing. Labeling is achieved either by fusing a biotin acceptor peptide to the POI and co-expressing BirA (Penalva and Keene, 2004; Jan et al., 2014; Williams et al., 2014) or by targeting APEX2/HRP to ribosomes (APEX-RIP; Kaewsapsak et al., 2017) or subcellular compartments (Proximity-CLIP; Benhalevy et al., 2018). High temporal resolution is a prerequisite for spatial transcriptomics, and can either be achieved by prior biotin-starvation or by using APEX2. These methods are therefore unlikely to be useful in plants at this time.

Noncrosslinking methods use the ability of some enzymes to directly label nucleotides for purification and identification. Among them are methods that use peroxidases or miniSOG (TSA-seq; Chen et al., 2018), APEX-seq (Fazal et al., 2019; Padron et al., 2019; Zhou et al., 2019; Tran et al., 2021), and CAP-seq (Wang et al., 2019b; Ding et al., 2020), which are unlikely to be readily applicable in plants. Methods using POI-fused bacterial DNA methyltransferases and m6A-specific antibodies to map protein–DNA interactions (DamID; van Steensel and Henikoff, 2000) and MadID (Sobecki et al., 2018), however, have been used in plants (Germann et al., 2006; Germann and Gaudin, 2011). Conceptually similar methods for RNA identification, employing Drosophila adenosine deaminase (TRIBE; McMahon et al., 2016) or *C. elegans* poly(U) polymerase (RNA tagging; Lapointe et al., 2015) should be adaptable for plant use as well. However, as labeling is based on direct interaction, they are less suitable for exploring organellar RNAs.

## Limitations, challenges, and considerations for (plant) PL

For all their advantages, PL methods also have limitations. First, PL is generally not proof of direct interaction. Since most PL enzymes release their substrate to diffuse away after activation, labeled proteins, RNAs, and DNA can either be directly or indirectly associated with the bait or be labeled stochastically because they share the same subcellular location as the bait. Accordingly, any potential PPI has to be confirmed through complementary approaches. To reduce the number of false positives from stochastic labeling it is important to include suitable controls, such as compartment-wide expression of the free PL enzyme, or a combination of similarly expressed unrelated baits. The latter strategy has the added benefit, that “control” samples are not merely used for background subtraction, but provide additional protein interaction data. Using a large number of baits in the same subcellular compartment can provide spatial information about protein distribution in microdomains and be used to build protein networks and identify functional modules through prey-centric correlation analysis (Go et al., 2021).

Furthermore, as all PL enzymes have intrinsic biases, and labeling depends on protein abundance, size, fold, and amino acid composition, labeling intensity cannot be used to unambiguously infer interaction strength. Small, tightly folded proteins, and proteins with few surface-exposed acceptor residues are less likely to be labeled. Similarly, RNAs and DNA regions that are covered by proteins or are depleted of modifiable nucleobases (e.g. telomeres), are inaccessible to PL. Position of the PL enzyme on the bait can also impair labeling of bait molecules if the enzyme is sterically hindered or the labeling radius is insufficient. For most PL enzymes, the exact labeling radius is unknown and may be influenced by experimental conditions, such as labeling time and temperature, presence and length of a flexible linker, local cellular environments (e.g. pH and ATP concentration), and concentration of quenchers and prey molecules (Choi-Rhee et al., 2004; Kim et al., 2018).

Too much labeling can also pose a problem, because biotinylation prevents tryptic and LysC digest at the modified position and can change the properties of internally labeled peptides, potentially interfering with detectability. Moreover, the biotin–avidin interaction is so strong that biotinylated peptides are hard to elute from streptavidin beads. Therefore, with standard protocols, few biotinylated peptides are detected. While it is rarely necessary to identify the exact biotinylation sites, doing so can provide valuable information in some cases (e.g. for protein and chromosome topology analysis, identification of surface exposed, or disordered domains). This issue can be overcome by different strategies: using antibodies for pulldowns (Udeshi et al., 2017; Kim et al., 2018), more stringent or alternative elution protocols (Lee et al., 2016; Arora et al., 2020; Kido et al., 2020), peptide-level AP (Kim et al., 2018, 2019; Kido et al., 2020), and cleavable biotin substrates for improved elution from streptavidin beads (Li et al., 2021a). The use of cleavable substrates has the added advantage of improving recovery of truly labeled proteins while background proteins that nonspecifically bind the streptavidin beads are retained.

Due to their considerable size (∼30 kDa), fusing PL enzymes to a POI or targeting peptides may cause issues with protein integrity, activity, and targeting. In such cases, choosing a smaller enzyme-like miniTurbo, microID, or ultraID or using an alternative targeting strategy may help. For example, Xiong et al. (2021) recently employed green fluorescent protein (GFP) nanobodies to target TurboID to GFP-tagged bait proteins. They found, however, that the use of a conditionally stabilized nanobody (unstable when the binding pocket is unoccupied), was critical for low background, as overabundance of TurboID leads to mislocalization and nonspecific labeling. Their strategy is particularly interesting, as it allows researchers to use existing reporter lines by crossing them with a nanobody-TurboID line.

In addition to general challenges, every experimental system and PL enzyme has their own limitations and not all enzymes work in all organisms. In the past, most experiments were done in (mammalian) cell culture, which is easy to manipulate. The need for biotin-phenol delivery and H_2_O_2_, as well as the abundance of endogenous peroxidases and generation of reactive oxygen species (ROS) as signaling molecule, has so far proven prohibitive for usage in plants. Only biotin ligases were successfully used. Unlike APEX2, biotin ligases do not directly label RNA and DNA, which limits the possible applications somewhat. In addition, they require longer labeling times, especially for application in whole organisms, and have poor temporal control due to constitutive activity at endogenous biotin levels. Controlling activity by biotin depletion, as can be done in mammalian cell culture, is not easily possible, as plants produce biotin and biotin synthesis mutants are generally not viable (Pommerrenig et al., 2013). Endogenous biotin levels are likely in the high nanometer to low micrometer range, with concentrations varying in different tissues (low in cotyledons, higher in cauline leaves, and siliques; Shellhammer and Meinke, 1990) and subcellular compartments (mostly free biotin in cytosol and bound biotin in mitochondria and chloroplasts; Baldet et al., 1993). Depending on the tissue, noninduced labeling can therefore be substantial (Mair et al., 2019). However, while this necessitates an additional biotin depletion step before streptavidin pulldowns, it will not be an issue for most experimental questions.

It should also be noted that PL enzymes are not well suited for experimental questions where enzyme activity varies between samples. For example, biotin ligase activity is temperature-dependent (negligible at 4°C) and organelle-dependent (Branon et al., 2018; Kido et al., 2020). Biotin and ATP availability may also differ between subcellular compartments. It is therefore important to carefully consider the setup of any PL experiment and to include appropriate controls (see “Box 1” for resources for planning a (plant) PL experiment). Considerations include general suitability of the method, choice of PL enzyme and promoter, fusion orientation, additional tags, treatment mode, protein purification, and quantification protocol (for a more in-depth discussion see Mair et al., 2019).

## Imagining the future of PL in plants

With the development of faster and more active BioID versions (Branon et al., 2018; Kido et al., 2020; Zhao et al., 2021) PL is slowly gaining traction in plant research. We expect PL to be a valuable addition to the plant biology toolbox, enabling new discoveries in many areas of plant research. It could, for example, be used to identify currently unknown signaling networks or expand on existing ones, gain insights in plant–microbe interactions, identify short-lived/unstable interactions such as kinase/phosphatase–substrate interactions, obtain insights into protein trafficking and degradation (e.g. identification of E3 ligases) and identify interactions with membrane- or other hard-to purify proteins. PL also opens up possibilities for characterizing subcellular proteomes on a fine scale, including compartments that are not purifiable (e.g. membrane-less organelles, microdomains, and membrane contact sites). Recently, Mergner et al. (2020) presented a tissue-level multi-omics (proteins, phospho-sites, and transcripts) atlas of Arabidopsis. It would be interesting to generate an analogous “subcellular proteome atlas” or to compare subcellular proteomes in different cell types and in response to different growth conditions.

While in the past, most PL experiments were done with over-expressed bait proteins, going forward we should move to using endogenous and cell type-specific promoters. scRNA-seq is now revealing new cell types and stages in developmental trajectories, which would remain undiscovered using bulk methods, underlining the importance of finer distinction between cells within a tissue. As transcript and protein abundance do not always correlate well, generating, and integrating comparable RNA- and protein-level datasets will provide valuable insights into RNA and protein regulation and could be used to identify master regulators of transcriptional reprogramming. Since single-cell proteomics does not yet have sufficient resolution to identify regulatory proteins that are typically low abundant, PL proteomics could be used to complement scRNA-seq data. For example, cell-type- and stage-specific promoters identified from scRNA-seq could be used to generate protein data from like cells through PL at organellar resolution.

Although TurboID and miniTurbo performed well under different experimental conditions in plants in recent studies, several things remain to be tested (see “Outstanding questions box”).

We imagine that in the future, PL applications in plants will further expand beyond mere PPI and proteome discovery. Several of the methods described in this review can likely be adapted for plant use. For example, the adaptation of CasID for discovery of DNA binding proteins could be relatively straightforward, but might require the use of a more active biotin ligase with low background activity (e.g. miniTurbo) or split-TurboID for improved sensitivity and specificity, respectively. Similarly, by using more active biotin ligases, methods for identifying RNA-bound proteins should be applicable in plants as well. Since biotin ligases do not label RNA or DNA directly, we will likely have to rely on methods involving crosslinking or DamID, MadID, and TRIBE for such purposes. Although APEX2 is currently not suited for protein or nucleotide labeling in living plants, EM methods using APEX2 are probably in reach as labeling happens in fixed tissue. Recently introduced alternative substrates, such as clickable alkyne-phenol (Li et al., 2020), could further make APEX2 more suitable for plants, although high background from endogenous peroxidases and H_2_O_2_ might remain a problem. With a shorter labeling radius, NEDDylation and PUP-IT would also be interesting alternatives for identification of direct PPIs. Since the neddylation pathway is present in plants (Mergner and Schwechheimer, 2014; Schwechheimer, 2018), it is likely that NEDDylation can be applied with relatively little modification, although it may be beneficial to adapt a plant E2 enzyme instead of Ubc12 to improve enzyme activity at plant growth temperatures and to add a second tag to NEDD8 for dual purification. Similarly, PUP-IT could be implemented by expressing both the PafA–POI fusion protein and bio-Pup in plant cells. Given that plants produce biotin, biotin supplementation for labeling of Pup might not be required. Whether PafA activity is sufficient for effective labeling under favorable plant growth temperatures remains to be seen.

**Advances**

- In recent years, PL enzymes and enzyme variants have been developed that are faster and better suited for use in plants.
- Increased sensitivity and temporal resolution of enzymes like TurboID and miniTurbo enable PL at cell type-specific level in living organisms.
- Development of new methods for microscopy and exploring protein–DNA/RNA interaction could broaden the spectrum of PL applications in plants.

Box 1 Resources for planning a PL experiment (in plants).

- Details on experimental planning and considerations are discussed in: Mair et al. (2019) and Zhang et al. (2020a).
- Extensive testing of optimal experimental conditions for TurboID and miniTurbo in different plant species can be found in: Mair et al. (2019), Zhang et al. (2019), and Arora et al. (2020).
- Protocols for PL in plants can be found in the corresponding publications (see main text and Supplemental Table S2) and Germann and Gaudin (2011), Kim et al. (2018), Kido et al. (2020), Zhang et al. (2020a), and Cho et al. (2020b).
- Plasmids available as a community resource: (1) Set of Gateway plasmids for TurboID and miniTurbo POI fusions and subcellular targeting, deposited with addgene (Mair et al., 2019); (2) Set of Gateway and GoldenGate plasmids for BioID, BioID2, TurboID, and miniTurbo POI fusions and subcellular targeting (codon optimized; Arora et al., 2020).

## Supplemental data 

The following materials are available in the online version of this article.

**Supplemental Table S1.** PL and split-PL enzymes.

**Supplemental Table S2.** List of plant PL experiments.

**Supplemental Table S3.** Methods for identification of protein–RNA/DNA interactions and subcellular RNA.

## Supplementary Material

kiab479_Supplementary_Data

## Notes

A.M. and D.C.B. wrote the article. A.M. agrees to serve as the author responsible for contact and ensures communication.

The author responsible for distribution of materials integral to the findings presented in this article in accordance with the policy described in the Instructions for Authors (https://academic.oup.com/plphys/pages/general-instructions) is: Andrea Mair (maira@stanford.edu).
