# Supplementary material for: Advances in enzyme-mediated proximity labeling and its potential for plant research
Source: Plant Physiol. 2021 Oct 18;188(2):756–68. doi: 10.1093/plphys/kiab479 (PMC8825456; doi:10.1093/plphys/kiab479)
Supplement: kiab479_Supplementary_Data [file kiab479_supplementary_data.pdf]

Supplemental Data

Supplemental Table S1: PL and split-PL enzymes

Enzyme name	Origin	Size (kDa)	Substrate/requirements for PL	Labeling time/conditions	Labeling radius	Used in plants?	Labels what?	References
HRP	Horseradish peroxidase	44	Various substrates (e. g. azide-biotin, biotin-phenol, …), H_2O_2, heme if used extracellularly	5 min – 2 h; oxidizing conditions	200-300nm (EMARS) < 200nM (SPPLAT)	NO	Proteins on Tyr, Trp, Cys, His; RNA/DNA on G	(Kotani et al., 2008; Rees et al., 2015)
APEX	Pea or soybean APX	28	Biotin-phenol, other substrates, H_2O_2	1 min	< 20 nm	NO	Proteins on Tyr, Trp, Cys, His; RNA/DNA on G	(Martell et al., 2012; Rhee et al., 2013)
APEX2	Soybean APX	28	Biotin-phenol, other substrates, H_2O_2	1 min	< 20 nm	NO	Proteins on Tyr, Trp, Cys, His; RNA/DNA on G	(Lam et al., 2015)
APEX2C32S	Soybean APX	28	Biotin-phenol, other substrates, H_2O_2	1 min	< 20 nm	NO	Proteins on Tyr, Trp, Cys, His; RNA/DNA on G	(Huang et al., 2019)
BioID	*E. coli* BirA	35	Biotin, (ATP)	16-24 h, 37°C optimum	~ 10 nm	YES	Proteins (Lys)	(Choi-Rhee et al., 2004; Roux et al., 2012)
BioID2	*Aquifex aeolicus* BirA	26	Biotin, (ATP)	16-24 h, 50°C optimum, works well at 37°C	≥ 10 nm	YES	Proteins (Lys)	(Kim et al., 2016)
BASU	*Bacillus subtilis* BirA	28	Biotin, (ATP)	≥ 1 min for RaPID, 12h for protein labeling	~ 10 nm?	NO	Proteins (Lys)	(Ramanathan et al., 2018)
TurboID	*E. coli* BirA (yeast evolution)	35	Biotin, (ATP)	≥ 10 min, 30-37°C optimum; works well at 20-25°C	≥ 35 nm, increasing with labeling times)	YES	Proteins (Lys)	(Branon et al., 2018)

miniTurbo	*E. coli* BirA (yeast evolution)	28	Biotin, (ATP)	≥ 10 min, 30°C optimum; works well at 20-25°C	≥ 10-35 nm?	YES	Proteins (Lys)	(Branon et al., 2018)
BirA (R118K)	*E. coli* BirA	35	Biotin, (ATP)	16-24h	~ 10 nm?	NO	Proteins (Lys)	(Oostdyk et al., 2019)
AirID	Synthetic BirA	35	Biotin, (ATP)	3-6h; works at 16-37°C	~ 10 nm?	NO	Proteins (Lys)	(Kido et al., 2020)
microID	*BioID2*	19.7	Biotin, (ATP)	≥ 1 h	~ 10 nm?	NO	Proteins (Lys)	(Zhao et al., 2021) preprint
ultraID	*BioID2*	19.7	Biotin, (ATP)	≥ 10 min	~ 10 nm?	NO	Proteins (Lys)	(Zhao et al., 2021) preprint
NEDDylation	Human Ubc12	21	His_6-biotin-NEDD8 (HB-NEDD8)	1-2 h in cell lysates, 48h in cells	Direct contact	NO	Proteins (Lys)	(Hill et al., 2016; Zhuang et al., 2013)
PUP-IT	*Corynebacterium glutamicum* PafA	51	Bio-PupE or bio-DE28	24 h	60-80 Å	NO	Proteins (Lys)	(Liu et al., 2018)
EXCELL (mgSrtA)	*S. aureus* SrtA	24	Biotin-LPETG (Biotin-LPXTG)	≥ 30 min	Direct contact	NO	Protein (N-terminal Gly)	(Ge et al., 2019)
miniSOG/SOPP2	LOV domain from *A. thaliana* phototropin 2	14/12	Various substartes (e. g. biotin-thiol, biotin-alkylamine, propargyl-amine, …)	~ 20-30 min	≤ 70 nm?	NO	Protein (Cys, (Lys, His, Ser)), RNA and DNA	(Ding et al., 2020; To et al., 2016; Wang et al., 2019)
Split-HRP (sHRP) (G213/N214)	HRP		Various substrates (e. g. azide-biotin, biotin-phenol, …), H_2O_2, heme if used extracellularly	≥ 1-10 min	< 20 nm	NO	Proteins on Tyr, Trp, Cys, His; RNA/DNA on G	(Martell et al., 2016)
Split-APEX2 (G201/L202)	Soybean APEX2		Biotin-phenol, other substrates, H_2O_2	≥ 1 min	< 20 nm	NO	Proteins on Tyr, Trp, Cys, His; RNA/DNA on G	(Xue et al., 2017)
Split-APEX2 (sAPEX2) (E200/G201)	Soybean APEX2		Biotin-phenol, other substrates, H_2O_2	≥ 1 min	< 20 nm	NO	Proteins on Tyr, Trp, Cys, His; RNA/DNA on G	(Han et al., 2019)
Split-BioID (E140/Q141)	BioID		Biotin, (ATP)	≥ 16h	~ 10 nm?	NO	Proteins (Lys)	(De Munter et al., 2017)

Split-BioID (E256/G257)	BioID		Biotin, (ATP)	≥ 20h	~ 10 nm?	NO	Proteins (Lys)	(Schopp et al., 2017)
ContactID (Split-BioID) (G78/G79)	BioID		Biotin, (ATP)	≥ 16h	~ 10 nm?	NO	Proteins (Lys)	(Kwak et al., 2020)
Split-TurboID (L73/G74)	TurboID		Biotin, (ATP)	≥ 1-4h	~ 10 nm?	NO	Proteins (Lys)	(Cho et al., 2020)
Split-miniSOG (G94/E95)	miniSOG		Various substartes (e. g. biotin-thiol, biotin-alkylamine, propargyl-amine, …)	<10 min	≤ 70 nm?	NO	Protein (Cys, (Lys, His, Ser)), RNA and DNA	(Boassa et al., 2019)

Supplemental Table S2: List of plant proximity labeling experiments

References	PL enzyme	Plant system	Promoter	Conditions	Baits
(Lo Presti et al., 2017)	BirA (codon optimized for plants)	Maize (stable expression)	maize UBQ promoter	No exogenous biotin, 4 days	pathogen effectors from the smut fungus *Ustilago maydis* (modified with the short peptide Avitag as biotinylation acceptor) > uptake into cytosol vs. apoplast
(Lin et al., 2017)	BioID [BirAG = BirA* with a cryptic intron site removed when expressed in rice and partial codon optimization)]	rice protoplasts (transient)	maize UBQ promoter	24h with 50µM biotin	OsFD1, OsFD2 (TF involved in vegetative growth in rice)
(Khan et al., 2018)	BioID	Arabidopsis (stable expression)	DEX inducible promoter	2 mM biotin, 24h	P. syringae effector protein HopF2
(Conlan et al., 2018)	BioID	*N. benthamiana* (transient)	35S promoter	75µM biotin, 24h	P. syringae effector protein AvrPto (to find immune system components at the PM)
(Das et al., 2019)	BioID	*N. benthamiana* (transient)	35S promoter	0.5mM biotin, 48h, 28C, TMV infection	TMV (tobacco mosaic virus) 126 kDa replicase
(Macharia et al., 2019)	BioID	*N. benthamiana* (transient)	35S promoter	2mM biotin, 3 days, TMV infection	core autophagy protein ATG8 during TMV infection
(Tang et al., 2020)	BioID2	Arabidopsis (stable expression)	35S promoter	50µM biotin, 24h	nuclear envelope TM proteins: ONM (WIP1, SINE1), INM (SUN1, NEAP1) and NPC (Nup93a, Nup82)

(Huang et al., 2020)	BioID2	Arabidopsis (stable expression)	35S promoter	50µM biotin, 16h RT	Nuclear envelope proteins: ONM (WIT1), INM (SUN1 (Tang data), NEMP_1); PUX5 (SUN1 interacting protein), RHD3 (ER protein)
(Zhang et al., 2019)	TurboID	*N. benthamiana* (transient)	AtUBQ10 promoter	200µM biotin, 12h at RT with p50 induction	N (TIR-NLR immune receptor that confers resistance to TMV)
(Mair et al., 2019)	TurboID (and miniTurbo)	*N. benthamiana* (transient) and Arabidopsis (stable lines)	AtUBQ10 promoter and endogenous cell-type specific promoter (FAMA)	50µM biotin, 30 min – 3h	Cytosolic and nuclear TbID/mTb for testing activity under different conditions and in different tissues; FAMA (late stomatal lineage specific TF)
(Arora et al., 2020)	TurboID (and miniTurbo)	Tomato hairy root culture, *N. benthamiana* (transient), Arabidopsis (stable lines)	35S promoter	Tomato: 50µM biotin, 2h/24h<br>N. benthamiana: 50µM/1mM biotin, 24h/15/30min<br>Arabdopsis: 10min 1h, 6h, 24h, 25C	Tomato: free PL enzymes<br>N. benthamiana: membrane receptor complexes – co-expression of known complex components<br>Arabidopsis: TPLATE complex
(Kim et al., 2019)	TurboID	*N. benthamiana* (transient), Arabidopsis (stable)	35S promoter	50µM, 3h	BIN2 interactors (neg regulator of BR signaling)
(Xu et al., 2021)	TurboID	Arabidopsis (stable)	Endogenous promoters	50µM, 6h	XPO4/5/7 (exportins)

Supplemental Table S3: Methods for identification of protein-RNA/DNA interactions and subcellular RNA

Method	Labels what?	PL enzyme	Targeting method	References
CasID	Protein at DNA	BioID	dCas9 + sgRNA	(Schmidtmann et al., 2016)
C-BERST	Protein at DNA	APEX2	dCas9 + sgRNA	(Gao et al., 2018)
GLoPro	Protein at DNA	APEX2	dCas9 + sgRNA	(Myers et al., 2018)
CAPLOCUS	Protein, long-range DNA- and RNA interactions at DNA	MS2-CP-APEX2	dCas9 + sgRNA w/ MS2 stem loops (X-linking)	(Qiu et al., 2019)

CAPTURE	Protein, long-range DNA and RNA interactions at DNA	BirA	dCas9-biotin acceptor peptide + sgRNA (x-linking)	(Liu et al., 2017)
ChromID	Proteins at methylated DNA/histones	BASU	eCR (engineered chromatin readers)	(Villasenor et al., 2020)
RapID	Proteins at RNA	BASU (BioID, BioID2)	BoxB stem loop + λN peptide fused to PL enzyme	(Ramanathan et al., 2018)
RBPL	Proteins at RNA	BASU, APEX2, (BioID)	BoxB stem loop + λN peptide fused to PL enzyme	(Lu and Wei, 2019)
RNA-BioID	Proteins at RNA	BioID	MS2 stem loop + MS2-CP-BioID	(Mukherjee et al., 2019)
	Proteins at RNA	APEX2	MS2 stem loop + MS2-CP-APEX2 and dCas13-APEX2 plus crRNAs	(Han et al., 2020)
CARPID	Proteins at RNA	BASU	dCasRx-BASU plus gRNA	(Yi et al., 2020)
CBRPP	Proteins at RNA	BioID2/TbID/BASU/APEX2	dPspCas13b-PL enzyme plus crRNA	(Li et al., 2021)
CRUIS	Proteins at RNA	PUP-IT (PafA)	dLwaCas13a-PafA plus crRNA plus Bio-PupE	(Zhang et al., 2020)
RPL	Proteins at RNA	APEX2	dPspCas13b-APEX2 plus crRNA	(Lin et al., 2020)
	Protein-bound RNAs	BirA	POI-BAP plus BirA	(Penalva and Keene, 2004)
Proximity-specific ribosome profiling	Local translatome	BirA	Ribosome-AviTag plus localized BirA	(Jan et al., 2014; Williams et al., 2014)
APEX-RIP	Local RNAs	APEX2, HRP	Targeting sequence/protein; x-linking with FA	(Kaewsapsak et al., 2017)
Proximity-CLIP	Local proteins, RNAs and binding sites on RNAs	APEX2	Targeting sequence/protein; x-linking with UV	(Benhalevy et al., 2018)
TSA-seq	Local DNA > distance mapping	HRP	antibodies	(Chen et al., 2018)
APEX-seq	Local proteins and RNAs	APEX2	Targeting sequence/protein	(Fazal et al., 2019; Padron et al., 2019; Zhou et al., 2019)
	Proteins, RNA and DNA	APEX2	Targeting sequence/protein	(Tran et al., 2021)
CAP-seq	Local RNAs	miniSOG	Targeting sequence/protein	(Wang et al., 2019)
		miniSOG	Targeting sequence/protein	(Ding et al., 2020)
DamID	Protein-bound DNA	E. coli Dam	Targeting protein	(van Steensel and Henikoff, 2000)
MadID	Protein-bound DNA	E. coli M.EcoGII	Targeting protein	(Sobecki et al., 2018)
TRIBE	Protein-bound RNA	Drosophila ADAR	Targeting protein	(McMahon et al., 2016)
RNA tagging	Protein-bound RNA	C. elegans PUP-2	Targeting protein	(Lapointe et al., 2015)

References

Arora, D., Abel, N.B., Liu, C., Van Damme, P., Yperman, K., Eeckhout, D., Vu, L.D., Wang, J., Tornkvist, A., Impens, F.*, et al.* (2020). Establishment of Proximity-Dependent Biotinylation Approaches in Different Plant Model Systems. The Plant cell **32**: 3388-3407.

Benhalevy, D., Anastasakis, D.G., and Hafner, M. (2018). Proximity-CLIP provides a snapshot of protein-occupied RNA elements in subcellular compartments. Nat Methods **15**: 1074-1082.

Boassa, D., Lemieux, S.P., Lev-Ram, V., Hu, J., Xiong, Q., Phan, S., Mackey, M., Ramachandra, R., Peace, R.E., Adams, S.R.*, et al.* (2019). Split-miniSOG for Spatially Detecting Intracellular Protein-Protein Interactions by Correlated Light and Electron Microscopy. Cell Chem Biol **26**: 1407-1416 e1405.

Branon, T.C., Bosch, J.A., Sanchez, A.D., Udeshi, N.D., Svinkina, T., Carr, S.A., Feldman, J.L., Perrimon, N., and Ting, A.Y. (2018). Efficient proximity labeling in living cells and organisms with TurboID. Nat Biotechnol **36**: 880-887.

Chen, Y., Zhang, Y., Wang, Y., Zhang, L., Brinkman, E.K., Adam, S.A., Goldman, R., van Steensel, B., Ma, J., and Belmont, A.S. (2018). Mapping 3D genome organization relative to nuclear compartments using TSA-Seq as a cytological ruler. J Cell Biol **217**: 4025-4048.

Cho, K.F., Branon, T.C., Rajeev, S., Svinkina, T., Udeshi, N.D., Thoudam, T., Kwak, C., Rhee, H.W., Lee, I.K., Carr, S.A.*, et al.* (2020). Split-TurboID enables contact-dependent proximity labeling in cells. Proceedings of the National Academy of Sciences of the United States of America, **117**: 12143-12154

Choi-Rhee, E., Schulman, H., and Cronan, J.E. (2004). Promiscuous protein biotinylation by Escherichia coli biotin protein ligase. Protein Sci **13**: 3043-3050.

Conlan, B., Stoll, T., Gorman, J.J., Saur, I., and Rathjen, J.P. (2018). Development of a Rapid in planta BioID System as a Probe for Plasma Membrane-Associated Immunity Proteins. Frontiers in plant science **9:** 1882.

Das, P.P., Macharia, M.W., Lin, Q., and Wong, S.M. (2019). In planta proximity-dependent biotin identification (BioID) identifies a TMV replication co-chaperone NbSGT1 in the vicinity of 126kDa replicase. Journal of proteomics **204**,: 103402.

De Munter, S., Gornemann, J., Derua, R., Lesage, B., Qian, J., Heroes, E., Waelkens, E., Van Eynde, A., Beullens, M., and Bollen, M. (2017). Split-BioID: a proximity biotinylation assay for dimerization-dependent protein interactions. FEBS Lett **591**: 415-424.

Ding, T., Zhu, L., Fang, Y., Liu, Y., Tang, W., and Zou, P. (2020). Chromophore-Assisted Proximity Labeling of DNA Reveals Chromosomal Organization in Living Cells. Angew Chem Int Ed Engl **59**: 22933-22937.

Fazal, F.M., Han, S., Parker, K.R., Kaewsapsak, P., Xu, J., Boettiger, A.N., Chang, H.Y., and Ting, A.Y. (2019). Atlas of Subcellular RNA Localization Revealed by APEX-Seq. Cell **178**: 473-490 e426.

Gao, X.D., Tu, L.C., Mir, A., Rodriguez, T., Ding, Y., Leszyk, J., Dekker, J., Shaffer, S.A., Zhu, L.J., Wolfe, S.A., *et al.* (2018). C-BERST: defining subnuclear proteomic landscapes at genomic elements with dCas9-APEX2. Nat Methods **15**: 433-436.

Ge, Y., Chen, L., Liu, S., Zhao, J., Zhang, H., and Chen, P.R. (2019). Enzyme-Mediated Intercellular Proximity Labeling for Detecting Cell-Cell Interactions. J Am Chem Soc **141**: 1833-1837.

Han, S., Zhao, B.S., Myers, S.A., Carr, S.A., He, C., and Ting, A.Y. (2020). RNA-protein interaction mapping via MS2- or Cas13-based APEX targeting. Proceedings of the National Academy of Sciences of the United States of America **117**: 22068-22079.

Han, Y., Branon, T.C., Martell, J.D., Boassa, D., Shechner, D., Ellisman, M.H., and Ting, A. (2019). Directed Evolution of Split APEX2 Peroxidase. ACS Chem Biol **14**: 619-635.

Hill, Z.B., Pollock, S.B., Zhuang, M., and Wells, J.A. (2016). Direct Proximity Tagging of Small Molecule Protein Targets Using an Engineered NEDD8 Ligase. J Am Chem Soc **138**: 13123-13126.

Huang, A., Tang, Y., Shi, X., Jia, M., Zhu, J., Yan, X., Chen, H., and Gu, Y. (2020). Proximity labeling proteomics reveals critical regulators for inner nuclear membrane protein degradation in plants. Nat Commun **11**: 3284.

Huang, M.S., Lin, W.C., Chang, J.H., Cheng, C.H., Wang, H.Y., and Mou, K.Y. (2019). The cysteine-free single mutant C32S of APEX2 is a highly expressed and active fusion tag for proximity labeling applications. Protein Sci **28**: 1703-1712.

Jan, C.H., Williams, C.C., and Weissman, J.S. (2014). Principles of ER cotranslational translocation revealed by proximity-specific ribosome profiling. Science **346**: 1257521.

Kaewsapsak, P., Shechner, D.M., Mallard, W., Rinn, J.L., and Ting, A.Y. (2017). Live-cell mapping of organelle-associated RNAs via proximity biotinylation combined with protein-RNA crosslinking. eLife **6**.

Khan, M., Youn, J.Y., Gingras, A.C., Subramaniam, R., and Desveaux, D. (2018). In planta proximity dependent biotin identification (BioID). Sci Rep **8**: 9212.

Kido, K., Yamanaka, S., Nakano, S., Motani, K., Shinohara, S., Nozawa, A., Kosako, H., Ito, S., and Sawasaki, T. (2020). AirID, a novel proximity biotinylation enzyme, for analysis of protein-protein interactions. eLife **9**.

Kim, D.I., Jensen, S.C., Noble, K.A., Kc, B., Roux, K.H., Motamedchaboki, K., and Roux, K.J. (2016). An improved smaller biotin ligase for BioID proximity labeling. Mol Biol Cell **27**: 1188-1196.

Kim, T.-W., Park, C.H., Hsu, C.-C., Zhu, J.-Y., Hsiao, Y., Branon, T., Xu, S.-L., Ting, A.Y., and Wang, Z.-Y. (2019). Application of TurboID-mediated proximity labeling for mapping a GSK3 kinase signaling network in Arabidopsis. BioRxiv, 636324.

Kotani, N., Gu, J., Isaji, T., Udaka, K., Taniguchi, N., and Honke, K. (2008). Biochemical visualization of cell surface molecular clustering in living cells. Proceedings of the National Academy of Sciences of the United States of America **105**: 7405-7409.

Kwak, C., Shin, S., Park, J.S., Jung, M., Nhung, T.T.M., Kang, M.G., Lee, C., Kwon, T.H., Park, S.K., Mun, J.Y.*, et al.* (2020). Contact-ID, a tool for profiling organelle contact sites, reveals regulatory proteins of mitochondrial-associated membrane formation. Proceedings of the National Academy of Sciences of the United States of America **117**: 12109-12120.

Lam, S.S., Martell, J.D., Kamer, K.J., Deerinck, T.J., Ellisman, M.H., Mootha, V.K., and Ting, A.Y. (2015). Directed evolution of APEX2 for electron microscopy and proximity labeling. Nat Methods **12**: 51-54.

Lapointe, C.P., Wilinski, D., Saunders, H.A., and Wickens, M. (2015). Protein-RNA networks revealed through covalent RNA marks. Nat Methods **12**: 1163-1170.

Li, Y., Liu, S., Cao, L., Luo, Y., Du, H., Li, S., Zhang, Z., Guo, X., Tian, W., Wong, C.C., et al. (2021). CBRPP: a new RNA-centric method to study RNA-protein interactions. RNA Biol, 1-14.

Lin, Q., Zhou, Z., Luo, W., Fang, M., Li, M., and Li, H. (2017). Screening of Proximal and Interacting Proteins in Rice Protoplasts by Proximity-Dependent Biotinylation. Frontiers in plant science **8**: 749.

Lin, X., Fonseca, M.A.S., Corona, R.I., and Lawrenson, K. (2020). In vivo discovery of RNA proximal proteins in human cells via proximity-dependent biotinylation. bioRxiv, 2020.2002.2028.970442.

Liu, Q., Zheng, J., Sun, W., Huo, Y., Zhang, L., Hao, P., Wang, H., and Zhuang, M. (2018). A proximity-tagging system to identify membrane protein-protein interactions. Nat Methods **15**: 715-722.

Liu, X., Zhang, Y., Chen, Y., Li, M., Zhou, F., Li, K., Cao, H., Ni, M., Liu, Y., Gu, Z., et al. (2017). In Situ Capture of Chromatin Interactions by Biotinylated dCas9. Cell **170**: 1028-1043 e1019.

Lo Presti, L., Zechmann, B., Kumlehn, J., Liang, L., Lanver, D., Tanaka, S., Bock, R., and Kahmann, R. (2017). An assay for entry of secreted fungal effectors into plant cells. The New phytologist **213**: 956-964.

Lu, M., and Wei, W. (2019). Proximity labeling to detect RNA-protein interactions in live cells. FEBS Open Bio **9**: 1860-1868.

Macharia, M.W., Tan, W.Y.Z., Das, P.P., Naqvi, N.I., and Wong, S.M. (2019). Proximity-dependent biotinylation screening identifies NbHYPK as a novel interacting partner of ATG8 in plants. BMC Plant Biol **19**: 326.

Mair, A., Xu, S.L., Branon, T.C., Ting, A.Y., and Bergmann, D.C. (2019). Proximity labeling of protein complexes and cell-type-specific organellar proteomes in Arabidopsis enabled by TurboID. eLife **8**.

Martell, J.D., Deerinck, T.J., Sancak, Y., Poulos, T.L., Mootha, V.K., Sosinsky, G.E., Ellisman, M.H., and Ting, A.Y. (2012). Engineered ascorbate peroxidase as a genetically encoded reporter for electron microscopy. Nat Biotechnol **30**: 1143-1148.

Martell, J.D., Yamagata, M., Deerinck, T.J., Phan, S., Kwa, C.G., Ellisman, M.H., Sanes, J.R., and Ting, A.Y. (2016). A split horseradish peroxidase for the detection of intercellular protein-protein interactions and sensitive visualization of synapses. Nat Biotechnol **34**: 774-780.

McMahon, A.C., Rahman, R., Jin, H., Shen, J.L., Fieldsend, A., Luo, W., and Rosbash, M. (2016). TRIBE: Hijacking an RNA-Editing Enzyme to Identify Cell-Specific Targets of RNA-Binding Proteins. Cell **165**: 742-753.

Mukherjee, J., Hermesh, O., Eliscovich, C., Nalpas, N., Franz-Wachtel, M., Macek, B., and Jansen, R.P. (2019). beta-Actin mRNA interactome mapping by proximity biotinylation. Proceedings of the National Academy of Sciences of the United States of America **116**: 12863-12872.

Myers, S.A., Wright, J., Peckner, R., Kalish, B.T., Zhang, F., and Carr, S.A. (2018). Discovery of proteins associated with a predefined genomic locus via dCas9-APEX-mediated proximity labeling. Nat Methods **15**: 437-439.

Oostdyk, L.T., Shank, L., Jividen, K., Dworak, N., Sherman, N.E., and Paschal, B.M. (2019). Towards improving proximity labeling by the biotin ligase BirA. Methods **157**: 66-79.

Padron, A., Iwasaki, S., and Ingolia, N.T. (2019). Proximity RNA Labeling by APEX-Seq Reveals the Organization of Translation Initiation Complexes and Repressive RNA Granules. Mol Cell **75**: 875-887 e875.

Penalva, L.O., and Keene, J.D. (2004). Biotinylated tags for recovery and characterization of ribonucleoprotein complexes. Biotechniques **37**: 604, 606, 608-610.

Qiu, W., Xu, Z., Zhang, M., Zhang, D., Fan, H., Li, T., Wang, Q., Liu, P., Zhu, Z., Du, D., *et al.* (2019). Determination of local chromatin interactions using a combined CRISPR and peroxidase APEX2 system. Nucleic Acids Res **47**: e52.

Ramanathan, M., Majzoub, K., Rao, D.S., Neela, P.H., Zarnegar, B.J., Mondal, S., Roth, J.G., Gai, H., Kovalski, J.R., Siprashvili, Z., *et al.* (2018). RNA-protein interaction detection in living cells. Nat Methods **15**: 207-212.

Rees, J.S., Li, X.W., Perrett, S., Lilley, K.S., and Jackson, A.P. (2015). Selective Proteomic Proximity Labeling Assay Using Tyramide (SPPLAT): A Quantitative Method for the Proteomic Analysis of Localized Membrane-Bound Protein Clusters. Curr Protoc Protein Sci **80**: 19 27 11-19 27 18.

Rhee, H.W., Zou, P., Udeshi, N.D., Martell, J.D., Mootha, V.K., Carr, S.A., and Ting, A.Y. (2013). Proteomic mapping of mitochondria in living cells via spatially restricted enzymatic tagging. Science **339**: 1328-1331.

Roux, K.J., Kim, D.I., Raida, M., and Burke, B. (2012). A promiscuous biotin ligase fusion protein identifies proximal and interacting proteins in mammalian cells. J Cell Biol **196**: 801-810.

Schmidtmann, E., Anton, T., Rombaut, P., Herzog, F., and Leonhardt, H. (2016). Determination of local chromatin composition by CasID. Nucleus **7**: 476-484.

Schopp, I.M., Amaya Ramirez, C.C., Debeljak, J., Kreibich, E., Skribbe, M., Wild, K., and Bethune, J. (2017). Split-BioID a conditional proteomics approach to monitor the composition of spatiotemporally defined protein complexes. Nat Commun **8**: 15690.

Sobecki, M., Souaid, C., Boulay, J., Guerineau, V., Noordermeer, D., and Crabbe, L. (2018). MadID, a Versatile Approach to Map Protein-DNA Interactions, Highlights Telomere-Nuclear Envelope Contact Sites in Human Cells. Cell reports **25**: 2891-2903 e2895.

Tang, Y., Huang, A., and Gu, Y. (2020). Global profiling of plant nuclear membrane proteome in Arabidopsis. Nat Plants **6**: 838-847.

To, T.L., Medzihradszky, K.F., Burlingame, A.L., DeGrado, W.F., Jo, H., and Shu, X. (2016). Photoactivatable protein labeling by singlet oxygen mediated reactions. Bioorg Med Chem Lett **26**: 3359-3363.

Tran, J.R., Paulson, D.I., Moresco, J.J., Adam, S.A., Yates, J.R., Goldman, R.D., and Zheng, Y. (2021). An APEX2 proximity ligation method for mapping interactions with the nuclear lamina. J Cell Biol **220**.

van Steensel, B., and Henikoff, S. (2000). Identification of in vivo DNA targets of chromatin proteins using tethered dam methyltransferase. Nat Biotechnol **18**: 424-428.

Villasenor, R., Pfaendler, R., Ambrosi, C., Butz, S., Giuliani, S., Bryan, E., Sheahan, T.W., Gable, A.L., Schmolka, N., Manzo, M., *et al.* (2020). ChromID identifies the protein interactome at chromatin marks. Nat Biotechnol **38**: 728-736.

Wang, P., Tang, W., Li, Z., Zou, Z., Zhou, Y., Li, R., Xiong, T., Wang, J., and Zou, P. (2019). Mapping spatial transcriptome with light-activated proximity-dependent RNA labeling. Nat Chem Biol **15**: 1110-1119.

Williams, C.C., Jan, C.H., and Weissman, J.S. (2014). Targeting and plasticity of mitochondrial proteins revealed by proximity-specific ribosome profiling. Science **346**: 748-751.

Xu, F., Jia, M., Li, X., Tang, Y., Jiang, K., Bao, J., and Gu, Y. (2021). Exportin-4 coordinates nuclear shuttling of TOPLESS family transcription corepressors to regulate plant immunity. The Plant cell **33**: 697-713.

Xue, M., Hou, J., Wang, L., Cheng, D., Lu, J., Zheng, L., and Xu, T. (2017). Optimizing the fragment complementation of APEX2 for detection of specific protein-protein interactions in live cells. Sci Rep **7**: 12039.

Yi, W., Li, J., Zhu, X., Wang, X., Fan, L., Sun, W., Liao, L., Zhang, J., Li, X., Ye, J., *et al.* (2020). CRISPR-assisted detection of RNA-protein interactions in living cells. Nat Methods **17**: 685-688.

Zhang, Y., Song, G., Lal, N.K., Nagalakshmi, U., Li, Y., Zheng, W., Huang, P.J., Branon, T.C., Ting, A.Y., Walley, J.W., *et al.* (2019). TurboID-based proximity labeling reveals that UBR7 is a regulator of N NLR immune receptor-mediated immunity. Nat Commun **10**: 3252.

Zhang, Z., Sun, W., Shi, T., Lu, P., Zhuang, M., and Liu, J.L. (2020). Capturing RNA-protein interaction via CRUIS. Nucleic Acids Res **48**: e52.

Zhao, X., Bitsch, S., Kubitz, L., Schmitt, K., Deweid, L., Roehrig, A., Barazzone, E.C., Valerius, O., Kolmar, H., and Béthune, J. (2021). ultraID: a compact and efficient enzyme for proximity-dependent biotinylation in living cells. bioRxiv.

Zhou, Y., Wang, G., Wang, P., Li, Z., Yue, T., Wang, J., and Zou, P. (2019). Expanding APEX2 Substrates for Proximity-Dependent Labeling of Nucleic Acids and Proteins in Living Cells. Angewandte Chemie **131**: 11889-11893.

Zhuang, M., Guan, S., Wang, H., Burlingame, A.L., and Wells, J.A. (2013). Substrates of IAP ubiquitin ligases identified with a designed orthogonal E3 ligase, the NEDDylator. Mol Cell **49**: 273-282.
